# The relationship between physical activity, working memory, and mathematics achievement on the basis of socioeconomic status: the mediating role of physical fitness level

**DOI:** 10.3389/fnbeh.2026.1795851

**Published:** 2026-03-12

**Authors:** Süreyya Babayoğlu, Muhammed Sıddık Çemç, Sinan Vural, Görkem Açar, Emine Dereli Kamçı, Atacan Babayoğlu

**Affiliations:** 1 Faculty of Sport Sciences, Hacettepe University, Ankara, Türkiye; 2 Faculty of Sport Sciences, Istanbul Rumeli University, Istanbul, Türkiye; 3 Department of Physical Education and Sports, Boğaziçi University, Istanbul, Türkiye; 4 Faculty of Sport Sciences, Istanbul Gelisim University, Istanbul, Türkiye; 5 Orhantepe Family Health Center, Republic of Türkiye Ministry of Health, Istanbul, Türkiye

**Keywords:** adolescents, cognitive performance, mathematics achievement, physical activity, physical fitness, socioeconomic status, working memory

## Abstract

**Background:**

Socioeconomic disparities influence both academic achievement and health-related behaviors during adolescence; however, the mechanisms linking physical activity, physical fitness, and cognitive functions—particularly working memory—to academic outcomes remain insufficiently understood. Therefore, this study investigated the relationships among socioeconomic status (SES), physical activity, physical fitness, working memory, and mathematics achievement in adolescents, with a specific focus on the mediating role of physical fitness.

**Methods:**

A cross-sectional study was conducted with 310 ninth-grade students from five secondary schools in Türkiye. SES was assessed using a composite index including parental education, occupation, income, and household characteristics. Physical activity, physical fitness, working memory, and mathematics achievement were evaluated using validated standardized measures. Structural equation modeling (SEM) was applied to examine direct associations and the mediating role of physical fitness.

**Results:**

Maternal and paternal education levels were positively associated with mathematics achievement, whereas only maternal education showed a significant association with physical activity. SES was not directly associated with either physical activity or mathematics achievement. Working memory significantly predicted mathematics achievement. Physical activity was positively associated with physical fitness, which in turn predicted working memory. Additionally, physical fitness partially mediated the relationship between physical activity and working memory.

**Conclusion:**

Although causal relationships cannot be inferred due to the cross-sectional design, the findings suggest that regular engagement in physical activity and the development of physical fitness may contribute to improved cognitive functioning and academic performance during adolescence. Interventions aimed at enhancing physical fitness and promoting equitable access to physical activity opportunities may support both cognitive and educational outcomes.

## 1 Introduction

Executive functions are defined as a set of higher-level cognitive processes that enable individuals to regulate purposeful behavior, manage information, and adapt to environmental demands (Zhou and Tolmie, 2024; Cortés Pascual et al., 2019). This cognitive structure consists of three core components: inhibition, cognitive flexibility, and working memory (Miyake et al., 2000). These components form the cognitive infrastructure necessary for effective learning processes and are closely associated with academic achievement (McClelland and Cameron, 2019). Working memory, in particular, has emerged in recent years as one of the key determinants of academic performance and cognitive development (Ludyga et al., 2022; Capio et al., 2023; Masini et al., 2023; Lin et al., 2023).

Working memory is central to complex cognitive processes such as learning, problem solving, and reasoning, thanks to its capacity to store and process information in the short term (Baddeley, 2003; Diamond, 2013). Mathematical proficiency is highly dependent on this cognitive capacity and involves processes such as multi-step operations, symbolic manipulation, numerical estimation, and problem solving (Swanson and Kim, 2007; Friso-van den Bos et al., 2013; Baki, 2014; Berkowitz et al., 2022). Success in mathematics requires individuals to both temporarily retain existing knowledge and effectively use this knowledge by integrating it with new inputs; which clearly demonstrates the central role of working memory (Swanson, 2004; Bisanz et al., 2005; Siegler and Booth, 2005; Tronsky, 2005; Raghubar et al., 2010; Finell et al., 2022). In this context, it has been reported that working memory is related not only to academic achievement but also to behavioral variables such as physical activity level (Strenze, 2007; Titz and Karbach, 2014).

The positive effects of physical activity (PA) on cardiovascular health, musculoskeletal development, and metabolic balance in children and adolescents have long been known (Ortega et al., 2008; Myers et al., 2015). However, in recent years, an increasing number of studies have drawn attention to the effects of PA on cognitive functions and brain structure (Esteban-Cornejo et al., 2017). In particular, regular PA has been shown to support white and gray matter integrity, increase synaptic plasticity, and enhance the functionality of brain regions associated with executive functions (Chaddock-Heyman et al., 2018). PF components such as cardiorespiratory fitness and agility have been reported to be positively associated with attention, processing speed, and working memory performance (Esteban-Cornejo et al., 2017; Liu et al., 2018; Ludyga et al., 2018; Westfall et al., 2018).

However, the PA–cognition relationship is a complex system shaped by the interaction of numerous environmental and individual factors, such as age, PA level, the cognitive domain being assessed, and SES, rather than a unidimensional structure (López-Vicente et al., 2017; Pontifex et al., 2019; Williams et al., 2019). Studies examining the relationship between SES and PA levels in the literature present conflicting findings. While some studies report that high SES increases PA participation (Bradley, 2020; Malmo et al., 2021), others suggest that SES has no significant effect on PA (Ke et al., 2022; Pearson et al., 2022). A significant portion of these differences are thought to be related to sociocultural variables such as parental education level (Ruedl et al., 2021).

Parental education level is one of the strong determinants of both children’s PA habits and academic achievement (Lambert et al., 2022; Aelenei et al., 2023; Taseer et al., 2023). It is noted that parents with higher educational levels allocate more resources and importance to activities that support their children’s cognitive and physical development (Ziegeldorf et al., 2024). Mathematics achievement, in particular, is closely related to parental educational level and the cognitive support provided in the home environment (Hanson and Chen, 2007; O’Donoghue et al., 2018; Eime et al., 2015). However, the effects of SES and parental education on academic achievement have mostly been addressed through direct relationships; indirect pathways between PA, PA, and cognitive processes have not been modeled in a sufficiently comprehensive manner (Craike et al., 2018; Tandon et al., 2021; Stalling et al., 2022).

It has been suggested that PF is one of the underlying mechanisms of physical activity’s effects on cognitive functions (Wilson et al., 2004). It is stated that regular PA creates biological and neurophysiological stimuli on the central nervous system by increasing cardiorespiratory capacity, muscle strength, and agility performance; these stimuli also support executive functions such as working memory (Petersen et al., 2020; Muñoz-Galiano et al., 2020; Pedersen et al., 2021). However, the extent to which physical activity mediates the relationship between physical activity and working memory has been addressed in a limited number of studies.

A central theoretical assumption of the present study is that physical fitness represents a biological adaptation pathway through which physical activity influences higher-order cognitive processes. Physical activity is a behavioral exposure, whereas physical fitness reflects accumulated physiological adaptations resulting from repeated activity stimuli. Regular moderate-to-vigorous physical activity induces systemic and neural adaptations including increased cerebral blood flow, angiogenesis, and improved cerebrovascular regulation. These adaptations enhance oxygen and glucose delivery to metabolically active brain regions, particularly the prefrontal cortex and hippocampus, which are critically involved in working memory processes.

In addition to neurophysiological explanations, accumulating evidence suggests that motor and psychomotor development constitutes an important behavioral pathway linking physical fitness to cognitive and academic outcomes. Psychomotor skills integrate perceptual, motor, and executive processes and contribute to the organization of attention, memory, and learning-related behaviors. Recent empirical findings indicate that motor coordination, balance, spatial orientation, and visuomotor integration are associated with literacy, numeracy, and general academic performance (Amorim et al., 2024; Agostino et al., 2023). Psychomotor development has also been shown to support concentration, memory processes, and executive functioning, suggesting that motor competence may facilitate cognitive processing efficiency during learning tasks (Amorim et al., 2024; Agostino et al., 2023). Therefore, physical fitness may influence working memory not only through biological adaptations but also through improvements in motor coordination and perceptual–motor integration mechanisms. Within this perspective, physical fitness can be conceptualized as both a physiological and a behavioral–developmental mediator between physical activity and higher-order cognitive functions.

Cardiorespiratory fitness has been associated with increased hippocampal volume, elevated brain-derived neurotrophic factor (BDNF) expression, and enhanced synaptic plasticity, all of which support memory encoding and maintenance. In addition, improved fitness levels are linked to more efficient neural network functioning, reduced neural noise, and faster neural transmission due to myelination processes. Consequently, physical activity is not expected to influence working memory directly in all cases; rather, its cognitive effects are hypothesized to emerge primarily through the physiological adaptations captured by physical fitness. Therefore, physical fitness is theoretically positioned as a mediator that translates behavioral activity exposure into neurocognitive outcomes.

Based on this neurophysiological framework, we hypothesized that physical activity would positively predict physical fitness, and physical fitness, in turn, would predict working memory performance.

Adolescence, particularly around the age of 15, is a critical developmental stage during which individuals experience rapid changes in both physical and cognitive development (Hernandez, 2014; Bakan Kalaycıoğlu, 2015; Gustafsson and Nilsen, 2016). During this period, significant advances in executive functions are observed alongside the maturation of the prefrontal cortex, synaptic pruning, and myelination processes (Pinquart and Ebeling, 2020). Therefore, examining the relationships between PA, PF, and working memory in the context of math achievement in this age group carries high scientific value from developmental and educational perspectives.

This study is based on the assumption that the relationships between physical activity, cognitive functions, and academic achievement constitute a multidimensional system that should be addressed at different theoretical levels rather than as a single-level, linear structure. The effects of socioeconomic and familial determinants on physical activity behavior and academic outcomes are shaped more at the environmental and behavioral levels; whereas the effects of physical activity on cognitive processes such as working memory are thought to emerge largely through physiological adaptation mechanisms. Therefore, examining these relationships separately in terms of both environmental-behavioral determinants and physiological-cognitive mechanisms will increase the interpretability and theoretical explanatory power of the findings.

**FIGURE 1:**
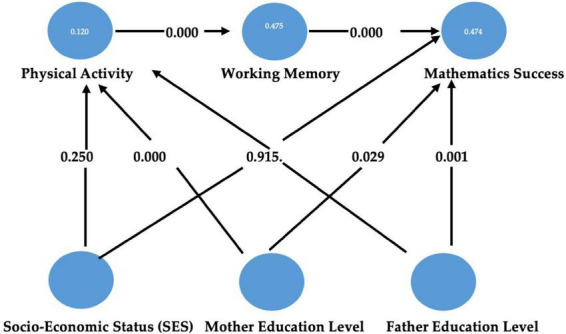
Structural equation modeling results for structural model 1.

Within this framework, the aim of the present study is to examine the relationships between physical activity, working memory, and mathematics achievement in relation to socioeconomic status by using structural equation modeling (SEM) to test a theoretically grounded mediation model in which physical fitness functions as a physiological intermediary between behavioral physical activity and working memory (Figure 1). The study seeks to address an important gap in the literature by integrating environmental (SES and parental education), behavioral (physical activity), physiological (physical fitness), and cognitive (working memory) variables within a comprehensive hierarchical model.

## 2 Materials and methods

### 2.1 Study design and participants

The research model was developed based on variables commonly used in the literature. In studies using structural equation modeling (SEM), the sample size depends on the number of variables in the model, the number of hypotheses, the number of paths, and the power of the analysis. In this study, the analyses were performed using the SmartPLS 4.1.0.5 software with the partial least squares (PLS) method. As stated by Chin (1998) and Hair et al. (2021), at least 150 participants are required for a reliable PLS-SEM analysis; 200 participants are recommended for more complex models (Memon et al., 2020). In this regard, the 310 participants included in the study were considered sufficient to increase the statistical power of the model, ensure generalizability, and obtain reliable results.

The proportional stratified sampling method was preferred for participant selection. This method ensures that subgroups in heterogeneous populations are included in the sample in proportion to their distribution in the population (Cochran, 1977; Creswell et al., 2007). It is an effective method, particularly for representing variables such as SES. 310 students in the 9th grade from five high schools located in different socioeconomic areas of Istanbul were included in the study on a voluntary basis. 160 of the participants were male (51.61%) and 150 were female (48.39%), indicating a balanced gender distribution. All participants were 15 years old (*M* = 15.0, SD = 0.0) and all were 9th grade students (Table 1). It was required that the participants did not have any health problems that would prevent them from participating in the PF tests with a doctor’s approval. This criterion was strictly observed in the study.

**TABLE 1:** Demographic profile of participants.

Variables	Category	*n*	%
Gender	Male	160	51.61
	Female	150	48.39
Age	15	310	100
Education level	9th grade	310	100
Maternal education level	Never attended school	3	0.97
	Primary school	67	21.61
	Middle school	70	22.58
	High school	113	36.45
	Associate degree	14	4.52
	Bachelor’s degree	33	10.65
	Master’s degree	10	3.23
Paternal education level	Never attended school	0	0.0
	Primary school	19	6.13
	Middle school	56	18.06
	High school	140	45.16
	Associate degree	9	2.9
	Bachelor’s degree	78	25.16
	Master’s degree	8	2.58
Socioeconomic status (SES)	A	53	17
	B	71	23
	C_1_	65	21
	C_2_	62	20
	D	59	19

To enhance representativeness, schools were selected from districts characterized by different socioeconomic profiles within the metropolitan area. The proportional stratified sampling strategy ensured that students from varying socioeconomic backgrounds were included in the sample according to their distribution in the population. All participants were enrolled in public secondary schools following the same national curriculum and educational regulations, which reduced variability related to institutional differences. Therefore, although the sample was limited to a single metropolitan region, the use of stratified sampling across multiple socioeconomic districts and a standardized national education system increases the external validity of the findings for urban adolescent populations in Türkiye.

### 2.2 Data collection instruments

#### 2.2.1 Demographic information form

A demographic information form was used to obtain participants’ age, gender, and class information.

#### 2.2.2 Health examination findings form

In the health examination findings form, a health examination was conducted by a family physician in accordance with World Health Organization (WHO) guidelines (World Health Organization, 2021) to assess the health status of participants and determine their eligibility for inclusion in the study. In this context, cardiovascular, respiratory, musculoskeletal, and general health status were evaluated. For detailed examination procedures, please refer to Supplementary material 1.

#### 2.2.3 Socio-economic status

In this study, SES was calculated using a multidimensional index formula developed by Kalaycıoğlu et al. (2010). The index includes indicators such as home and vehicle ownership, durable consumer goods, parental education level, and household income. These components were weighted to create an individual SES score. In the original study, individuals were classified into four categories (A: high, D: low SES) based on their total SES score. However, in this study, this classification was used only in descriptive analyses; in structural equation modeling, it was included in the model as a continuous and observed variable, not as a categorical variable. The calculation process and components of the SES variable are presented in detail in Supplementary material 2.

#### 2.2.4 Physical fitness

Participants’ PF levels were assessed using the ALPHA test battery, which includes components of cardiorespiratory endurance, muscle strength, and speed-agility (Ruiz et al., 2011). The ALPHA battery is a standard measurement tool that provides a valid and reliable measure of PF in adolescents.

Cardiovascular fitness was measured using the 20-meter shuttle run test, and the number of laps completed was recorded. The validity of the test shows a high correlation with VO2max values measured in a laboratory setting (*r* = 0.70–0.85), and test-retest reliability has been reported as ICC > 0.90 (Léger et al., 1988).

Muscle strength, standing long jump (lower extremity), and handgrip strength (upper extremity) tests were used to assess strength. The validity coefficients of the standing long jump test were reported as *r* = 0.80–0.90, while the reliability coefficients were ICC > 0.90 and Cronbach’s α = 0.85–0.95 (Ruiz et al., 2011; Castro-Piñero et al., 2010). Students performed three trials, and the longest jump was recorded in centimeters. Upper extremity strength was measured using a digital hand dynamometer (TKK 5101 Grip D, Takei, Tokyo, Japan); two trials were performed for each hand, and the average was taken. Muscle strength data were calculated in both relative (kg/kg) and absolute (cm⋅kg) values. The validity of the handgrip test was *r* = 0.80–0.90, and its reliability was ICC ≈ 0.95.

Speed and agility were measured using the 4 × 10 m shuttle run test, with the shortest time recorded in seconds. The test’s validity coefficients are *r* = 0.70–0.80, and its reliability is ICC ≈ 0.85 (Ortega et al., 2008). The completion time was reversed so that higher values indicate better performance.

In the structural equation model, PF was modeled as a formative latent construct. The subcomponents of cardiorespiratory fitness, muscle strength, and agility were included in the model as separately observed variables. A single total score was not calculated; the contribution of each test to the construct was analyzed through VIF and factor loadings (Table 2).

**TABLE 2:** Formative external model evaluations.

Variable	Indicator	Outer model VIF	Factor weights	Factor weights *P*-values	Factor loading	Factor loading *p-*value
Physical fitness	20 m shuttle run	1.025	0.297	0.014	0.427	0.004
	4 × 10 m shuttle run	1.055	0.094	0.360	0.310	0.030
	Handgrip strength	1.045	0.655	0.000	0.740	0.000
	Standing long Jump	1.040	0.548	0.000	0.657	0.000

#### 2.2.5 International physical activity questionnaire

To assess participants’ physical activity (PA) levels, the Turkish version of the International Physical Activity Questionnaire—Short Form (IPAQ) was used (Öztürk, 2005). This form, consisting of seven questions, assesses the time spent walking, engaging in moderate and vigorous physical activities, and sitting over the past 7 days. The total score was calculated by multiplying the time spent (minutes) and frequency (days) for walking, moderate, and vigorous physical activities, and energy expenditure was expressed in MET-minutes.

The standard MET coefficients used are: sitting = 1.5, walking = 3.3, moderate PA = 4.0, vigorous PA = 8.0. The validity and reliability of the IPAQ among high school students has been supported by studies conducted in different countries. Comparisons with objective measurement tools (e.g., accelerometers) show that the questionnaire provides moderate validity in measuring PA levels (*r* = 0.30–0.40) (Craig et al., 2003; Hagströmer et al., 2006). Reliability analyses using the test-retest method found ICC values in the range of 0.70–0.80 and demonstrated that the questionnaire is a consistent measurement tool (Hagströmer et al., 2006).

Although the IPAQ-SF is widely used in adolescent populations, self-report measures may be affected by recall errors and social desirability bias. To minimize these risks, several precautions were implemented. First, questionnaires were administered in a supervised classroom setting, and standardized instructions were read aloud to ensure a common understanding of activity intensity levels (walking, moderate, and vigorous). Second, examples of typical daily activities relevant to adolescents (e.g., physical education classes, organized sports training, active commuting, and recreational play) were provided to facilitate accurate recall. Third, participants were explicitly informed that the responses would be anonymized and would not affect their academic evaluation, reducing pressure to overreport activity levels. Researchers remained present during completion to clarify questions but avoided leading responses. In addition, the recall period was restricted to the previous 7 days, which is recommended to reduce memory bias in adolescent populations. These procedures were applied to improve response accuracy and reduce systematic reporting bias.

#### 2.2.6 Working memory

Participants’ working memory capacities were measured using the Brain Workshop (v4.8.1) software based on the n-back task. The Turkish adaptation of this open-source software, developed using the Python programing language, was carried out by Altun and Çevik (2012). The reliability coefficients of the n-back tasks were reported in the range of *r* = 0.70–0.85, and their reliability was supported by test-retest methods (Jaeggi et al., 2010). The application was performed in a quiet environment using a laptop and headphones in full-screen mode.

The tasks were presented in three formats: visual, auditory, and visual + auditory. Initially, participants completed only the 1-back task sets. At this stage, participants were expected to match by keeping the previous position or sound in memory. The “A” key was pressed for visual matching and the “L” key for auditory matching. Both keys were used as appropriate in triple (location + sound) matching. After the practice phase was completed, the participants moved on to the 2-back task set. At this stage, participants matched stimuli two steps prior. The response time was kept constant at 3.5 s for each stimulus. Each task set consisted of 24 trials lasting 84 s. Each task type was administered only once during the test; visual and auditory 2-back task performances were recorded as scores representing participants’ working memory capacity. The total administration time was approximately 1 h per participant. To prevent cognitive fatigue, the tasks were presented gradually and intermittently, starting at the 1-back level, with 2–3 min breaks between each task set. Observations indicated that participants’ attention levels were maintained throughout the test, and no significant decline in performance was observed.

Because the n-back assessment requires sustained attention, precautions were taken to prevent cognitive fatigue from influencing performance. The task was administered individually in a quiet room with minimal external distractions. The protocol followed a progressive structure beginning with 1-back practice trials to ensure task familiarization before the 2-back condition. Short rest intervals (2–3 min) were provided between each task block, and participants were encouraged to relax their eyes and hands during breaks. The total active task time was substantially shorter than the total session duration, as breaks and instructions constituted a significant portion of the session. Moreover, performance patterns were monitored during testing; no systematic decline in response accuracy or reaction time across blocks was observed, suggesting that fatigue did not meaningfully affect working memory scores. These procedures are consistent with cognitive testing recommendations for adolescent populations and were implemented to preserve measurement validity.

#### 2.2.7 Mathematics achievement

Participants’ mathematics achievement levels were assessed using the results of the 2024 High School Transition Exam mathematics test. The LGS is a standardized and centralized exam system administered simultaneously in 81 provinces and overseas exam centers by the Ministry of National Education of the Republic of Turkey. The exam consists of two sections: verbal and numerical. The mathematics test in the numerical section aims to measure students’ higher-order cognitive skills such as reading comprehension, problem solving, analysis, drawing conclusions, and critical thinking (Millî Eğitim Bakanlığı, 2017).

### 2.3 Data collection process

The data collection process began with obtaining the necessary institutional permissions. Official approvals were obtained from school administrators, participants and their parents were informed, and written consent forms were collected. The applications were carried out during class hours under teacher supervision. Prior to the PF assessment, students were examined by their family doctor according to WHO criteria. Only individuals who were medically fit were included in the study. In the first phase, students were given a demographic information form and the IPAQ, while parents were given the SES form. Researchers provided support during the form completion process. The working memory test was administered one-on-one in a quiet environment during the second week. Breaks were given between tasks; the application lasted approximately 1 h for each student. All tests were completed within 4–6 days. PF tests were administered on different days under the supervision of a doctor and completed within a week. Mathematics achievement data were obtained from the 2024 LGS results. As the study was conducted with 15-year-old students, special attention was paid to ethical principles. The physical and cognitive limitations of the participants were taken into account; it was clearly stated that they had the right to leave the study at any time. The research process was conducted within the framework of the Helsinki Declaration and national ethical rules and was approved by the Istanbul Rumeli University Ethics Committee (April 26, 2024, Item No. 3).

### 2.4 Data analysis

Research data were analyzed using SPSS 28.0 (IBM Corp., Armonk, NY, United States) software. Descriptive statistics were calculated for the participants’ demographic characteristics. Hypothesis testing and structural modeling analyses were performed using the SmartPLS 4.1.0.5 software with the Partial Least Squares (PLS-SEM) method. This method is preferred in complex models, small sample sizes, and non-normally distributed data. Mathematics achievement was used as the dependent variable; the structural model was created to examine the effects of variables such as PA, PF, working memory, SES, and parental education. Only one latent variable, PF, was modeled in a formative structure. The PF components consisted of a 20-meter shuttle run, a 4 × 10-meter agility test, a standing long jump, and a handgrip strength test. The other variables (PA, working memory, math achievement, SES, and parental education) were analyzed as observed variables. The mediating effect of PF between PA and working memory was tested using the bootstrap method with 5,000 resamples. Indirect path coefficients and significance levels were calculated. In the evaluation of the formative structure, outer weight, outer loading, and VIF values were examined; no multicollinearity problem was found (Browne and Cudeck, 1993; Hanafiah, 2020; Hair et al., 2021). Parental education level was included in the model as a separate variable from the SES index. This distinction was made to ensure that parental education directly affects children’s academic achievement, while SES represents more general socioeconomic conditions. VIF values below 3.0, indicated that the two variables contributed independently to the model. The overall fit of the model was tested using the SRMR, d_ULS, d_G, χ^2^, and NFI indices; all values were found to be acceptable. These findings indicate that the model’s fit is adequate and its predictive power is high (Browne and Cudeck, 1993).

In formative measurement models, indicators are not expected to be interchangeable, and a non-significant outer weight does not automatically justify removal of an indicator. According to PLS-SEM guidelines, an indicator may be retained when its outer loading remains acceptable and when it represents a theoretically essential component of the construct. Physical fitness is a multidimensional construct comprising cardiorespiratory endurance, muscular strength, and motor performance capacities such as agility. The 4 × 10 m shuttle run was retained because it uniquely captures neuromotor coordination, speed of direction change, and perceptual-motor integration, which are not represented by aerobic capacity or strength measures.

Furthermore, multicollinearity diagnostics were examined using VIF values, and all indicators were below the recommended threshold (VIF < 3.0), indicating that the non-significant outer weight was not due to redundancy with other indicators. Instead, it suggests that agility contributes complementary rather than overlapping information to the physical fitness construct. Removing this indicator would reduce the conceptual completeness of the physical fitness variable and bias the construct toward purely physiological components.

## 3 Results

First, descriptive statistics for the participants’ scores were presented. Subsequently, since the variables included in the model were formative constructs, the findings related to the measurement models—including outer VIF (Variance Inflation Factors) values, factor weights, and model fit indices—were reported to assess validity. Finally, the structural relationships among the variables were defined, and the hypothesized relationships were tested.

### 3.1 Preliminary analysis

The findings revealed that participants scored an average of 2.03 ± 0.76 for PA and 53.59 ± 12.26 for PF, indicating a moderate level of participation in PA and a reasonable level of PF. Moreover, the results showed considerable variability in working memory (46.14 ± 13.67) and mathematics achievement (52.94 ± 18.41). The socioeconomic indicators (54.90 ± 13.69) exhibited a relatively heterogeneous distribution within the sample. In contrast, the low variance in maternal education level (2.50 ± 1.12) and paternal education level (2.80 ± 1.15) suggests that participants had a relatively homogeneous educational background (Table 3). These findings support the conceptual and statistical distinction between parental education levels and the SES index. Both sets of variables represent different dimensions of socioeconomic status—with parental education reflecting individual cognitive–cultural capital, and SES representing environmental and economic resources.

**TABLE 3:** Descriptive statistics of the constructs.

Construct	Mean ± SD
Physical activity	2.03 ± 0.76
Physical fitness	53.59 ± 12.26
a. Handgrip strength	29.91 ± 7.83
b. Standing long jump	154.60 ± 31.06
c. 4 × 10 m shuttle run	-12.21 ± 2.79
d. 20 m shuttle run	40.06 ± 7.37
Working memory	46.14 ± 13.67
Math achievement	52.94 ± 18.41
Mother’s education level	2.50 ± 1.12
Father’s education level	2.80 ± 1.15
Socioeconomic status (SES)	54.90 ± 13.69

### 3.2 Measurement validity

Both models in this study included only formative constructs. In formative structures, validity—rather than reliability—is evaluated, since reliability coefficients are not applicable (Hanafiah, 2020). For validity assessment, outer VIF values and factor weights were examined. First, outer VIF coefficients were calculated. According to established criteria, outer VIF values should be below 5 (Hair et al., 2017), and as shown in Table 2, all values met this requirement. Next, for assessing the validity of formative constructs, the *p*-values of the factor weights (outer weights) were checked to determine whether they were below 0.05 (*p* ≤ 0.05) (Hair et al., 2012). When the *p*-values of the indicators’ factor weights were examined (Table 2), it was observed that one indicator (4 × 10 m shuttle run) had a *p* > 0.05. However, its factor loading (outer loading) was found to be below 0.05, confirming its significance. Since the other variables were represented by single indicators, factor weights and loadings were not calculated for them. Therefore, it was concluded that the validity of all formative constructs included in the model was adequately established.

The non-significant outer weight of the shuttle-run indicator should not be interpreted as a lack of relevance. In formative constructs, statistical significance reflects relative contribution within the specific sample rather than conceptual importance. The acceptable loading value and absence of multicollinearity indicate that agility performance provides distinct variance within the physical fitness construct. Therefore, the indicator was retained to preserve construct validity and theoretical coverage.

To assess the fit of Models 1 and 2, the following indices were calculated: standardized root mean square residual (SRMR), unweighted least squares discrepancy (d_ULS), geodesic discrepancy (d_G), chi-square (χ^2^), and the normed fit index (NFI) (Barrera et al., 2022). The computed values were found to be within acceptable ranges and thresholds (Table 4). These model fit calculations provided values that supported the adequacy and acceptance of the models within the framework of structural equation modeling (SEM) (Browne and Cudeck, 1993).

**TABLE 4:** Model fitness for M1 and M2.

Fit indices	Structured model 1	Structured model 2
Standardized Squared Mean Squared Residual (SRMR)	0.054	0.031
Unweighted Minimum Squares Discrepancy (d_ULS)	0.060	0.020
Geodetic Discrepancy (d_G)	0.014	0.013
Chi-Square	7.236	6.703
Normed Fit Index (NFI)	0.967	0.951

### 3.3 Hypothesis testing

**FIGURE 2:**
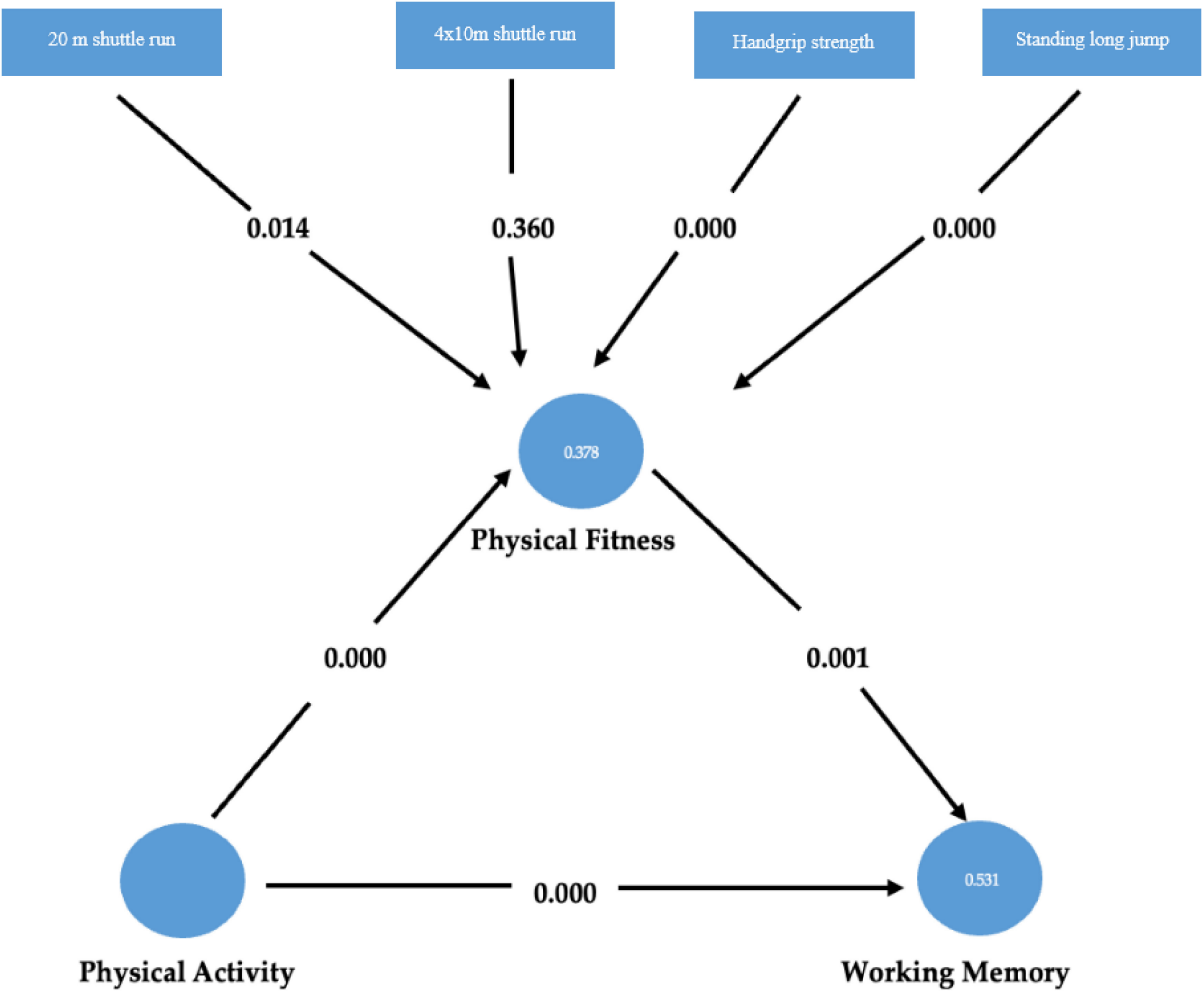
Structural equation modeling results for structural model 2.

The proposed hypotheses were analyzed using the SmartPLS 4.1.0.5 statistical package. In the SEM analysis, path coefficients (β), standard deviations, *p*-values, and *t*-values were calculated to determine whether each hypothesis was accepted or rejected (Table 5). In addition to the seven direct hypothesized paths, four mediating paths were tested (Figure 2). The path coefficient represents the effect of a predictor on the outcome variable (i.e., the change in the outcome for each one-unit change in the predictor), while the level of significance is determined based on the associated *p*-value.

**TABLE 5:** SEM results.

Path coefficients	Original sample (O)	Standard deviation (STDEV)	T statistics (| O/STDEV|)	*p*-values
**Structural model 1**				
SES → PA	-0.155	0.135	1,151	0.250
SES → MS	-0.013	0.119	0.107	0.915
MEL → PA	0.364	0.101	3.602	0.000
FEL → PA	0.080	0.105	0.764	0.445
MEL → MS	0.198	0.091	2.179	0.029
FEL → MS	0.273	0.079	3.443	0.001
WM → MS	0.663	0.071	9.394	0.000
**Structural model 2**				
PA → WM (First stage)	0.689	0.048	14.250	0.000
PA → WM (Second stage)	0.505	0.080	6.278	0.000
PA → PF	0.615	0.065	9.524	0.000
PF → WM	0.300	0.086	3.478	0.001
PA → PF → WM	0.184	0.061	3.033	0.002

In Structural Model 1, seven direct hypothesized paths were tested (Table 5). The SEM results indicated that the SES index did not positively affect either PA level (β = –0.155, *T* = 1.151, *p* > 0.05) or mathematics achievement (β = –0.013, *T* = 0.107, *p* > 0.05), thus H1 and H2 were not supported. The maternal education level positively affected PA level (β = 0.364, *T* = 3.602, *p* < 0.05), supporting H3, whereas the paternal education level did not have a significant positive effect on PA level (β = 0.080, *T* = 0.764, *p* > 0.05), so H4 was not supported. Both maternal education level (β = 0.198, *T* = 2.179, *p* < 0.05) and paternal education level (β = 0.273, *T* = 3.443, *p* < 0.05) were found to positively affect mathematics achievement, supporting H5 and H6. Finally, working memory was observed to positively affect mathematics achievement (β = 0.663, *T* = 9.394, *p* < 0.05), providing support for H7.

In Structural Model 2, PF was introduced as a mediating variable, comprising four mediating hypothesized paths (Table 5). In the first stage of the mediation analysis, the mediating variable (PF level) was removed from the model, and the significance of the path coefficients was tested. The effect of PA level on working memory was found to be statistically significant (β = 0.689, *T* = 14.250, *p* < 0.05), thus supporting H8. In the second stage, the mediating variable (PF) was reintroduced into the model, and the significance of the path coefficients was re-evaluated. The effects of PA level on PF level (β = 0.505, *T* = 6.278, *p* < 0.05) and of PF level on working memory (β = 0.300, *T* = 3.478, *p* < 0.05) were both found to be significant, supporting H9 and H10. Structural model analyses were conducted using SmartPLS 4, and the significance of indirect effects was tested through the bootstrapping method with 5,000 samples and a 95% confidence interval (Preacher and Hayes, 2008). The evaluation of mediation followed the Baron and Kenny (1986) approach, as it is theory-driven and suitable for explaining hierarchical relationships within structural models. Following the identification of mediation, the Variance Accounted For (VAF) values were calculated. The VAF for the PA → PF → Working Memory pathway was 0.21, indicating that PF level partially mediates the relationship between PA level and working memory. According to the classification proposed by Hair et al. (2021), a VAF of 0.21 reflects a weak partial mediation effect. These findings suggest that although the mediation effect is statistically significant, the indirect effect remains limited in magnitude. Thus, the mediating role of PF is not strong, and the direct effect of PA on working memory remains clearly evident in the model, thereby supporting H11.

As presented in Table 6, the structural model demonstrated a satisfactory level of explanatory power for the main latent variables. The coefficients of determination (R^2^) indicated that 52% of the variance in PF, 48% of the variance in working memory, and 45% of the variance in mathematics achievement were explained by the predictor variables included in the model. According to the evaluation criteria proposed by Hair et al. (2021), these R^2^ values represent moderate to high predictive power. These findings indicate that the model effectively captures the multidimensional interaction among PA, PF, and cognitive–academic performance, and that the proposed mediation structure is quantitatively supported.

**TABLE 6:** Coefficient of determination (R^2^) values.

Variable	*R* ^2^
Physical fitness	0.52
Working memory	0.48
Mathematics success	0.45

Although the indirect effect of physical activity on working memory through physical fitness was statistically significant, its magnitude was limited. The calculated Variance Accounted For (VAF) value was 0.21, indicating a weak partial mediation. This finding shows that the majority of the association between physical activity and working memory is explained by the direct pathway rather than the mediating pathway through physical fitness. Therefore, physical fitness contributes to the model as a complementary explanatory factor rather than a dominant mechanism linking physical activity to cognitive performance.

## 4 Discussion

Using SEM, this study is among the first in this age group and cultural context to examine the associations among SES, PA, working memory, and mathematics achievement, with PF modeled as a mediator. The findings are discussed below under six main themes and compared with the relevant literature.

### 4.1 SES and PA

SES was not found to positively influence PA. Although this result diverges from studies reporting higher PA levels among individuals with higher SES (Ke et al., 2022; Gidlow et al., 2006, Stalsberg and Pedersen, 2010), it is consistent with findings indicating no significant effect of SES on PA (Trost et al., 2002; Beenackers et al., 2012; Heradstveit et al., 2020; Falese et al., 2021) The results suggest that PA may be shaped less by economic resources than by environmental and cultural factors, social support, norms, and time constraints; therefore, sociocultural mechanisms such as social context and school environment may play a more decisive role in the SES–PA relationship. Accordingly, future studies should model these mechanisms as mediating variables.

However, the absence of a statistically significant SES effect should be interpreted cautiously. The participants were drawn from a relatively homogeneous educational context, consisting of students of the same age group and grade level within a single metropolitan area. Although schools were selected from different districts, the variability of socioeconomic conditions may have been narrower than in nationally representative samples. Restricted variance in SES can attenuate observable associations in structural models and may reduce statistical power to detect contextual effects. Therefore, the present findings do not necessarily indicate that SES has no influence on physical activity or academic achievement; rather, they suggest that SES-related effects were not detectable within this specific sample structure.

### 4.2 Parental education and PA

Maternal education level was found to positively influence PA, whereas paternal education level did not show a significant effect on PA. This finding is consistent with studies indicating that maternal education, in particular, is associated with higher PA levels in children (van Ansem et al., 2014; Maric et al., 2020). Mothers’ more active involvement in children’s daily routines and leisure-time activities may strengthen support and guidance for PA as educational attainment increases. Accordingly, PA behavior appears to be shaped not only by socioeconomic conditions but also by parents’ education-related awareness and supportive roles. In addition, parental education level has been reported to positively contribute to mathematics achievement (Hill and Tyson, 2009).

### 4.3 SES and academic achievement

Another key finding of the study is that SES did not directly affect mathematics achievement. Although SES has long been emphasized as an important correlate of academic achievement (Sirin, 2005; Reardon, 2018; Bradley and Corwyn, 2002; Duncan and Magnuson, 2012), prior research also indicates that this relationship is not always direct (Erola et al., 2016; Thomson, 2018; Vadivel et al., 2023). The present results suggest that mathematics achievement may be more strongly shaped by cognitive characteristics, teacher quality, and educational context, as well as parental education and cognitive support in the home environment. Indeed, the role of cognitive abilities and genetic factors in mathematics achievement has been highlighted (Peng and Kievit, 2020), with genetic influences reported to be more predictive than SES (Tucker-Drob and Bates, 2016). Moreover, the effects of educational strategies and individual processes (e.g., learning approaches, self-efficacy, and motivation) (Dolu, 2020; Olsen and Huang, 2021), together with the practice-intensive nature of mathematics learning (Boaler, 2022), indicate that the influence of SES on mathematics achievement may operate primarily through indirect mechanisms.

### 4.4 PA and working memory

This study found that PA positively influenced working memory. The findings are consistent with prior evidence indicating that PA supports neurocognitive processes (Cotman and Berchtold, 2002; Stillman et al., 2016; Mandolesi et al., 2018). This effect may be explained by the cognitive reserve hypothesis, whereby increased neuronal resilience enhances working memory capacity (Stern, 2009), as well as by the aerobic fitness hypothesis, which posits that improved cardiovascular fitness supports neurogenesis and synaptic plasticity, thereby strengthening prefrontal cortex functioning (Erickson et al., 2015). In addition, PA may affect cognitive performance indirectly by reducing fatigue (Ehlers et al., 2017). Nevertheless, it should be noted that this effect may also be shaped by motivational and psychosocial components.

### 4.5 PA and PF

PA was found to have a direct and significant effect on PF, consistent with previous research (Warburton et al., 2006; Haskell et al., 2007; World Health Organization, 2010; Booth et al., 2012). PA and PF represent mutually reinforcing constructs, whereby regular PA enhances PF, and higher levels of PF may, in turn, facilitate greater engagement in PA. Given that PF comprises multiple components—such as cardiorespiratory fitness, muscular strength, and agility—the relationships between PA and these subcomponents are discussed below in light of the present findings.

Cardiorespiratory fitness refers to the capacity to supply oxygen to the body during exercise (Myers et al., 2002; Ross et al., 2016). Regular PA is known to enhance aerobic capacity (Kao et al., 2020), and the present study showed that PA particularly supports cardiorespiratory fitness in adolescents. However, differences in PF levels may be influenced not only by PA but also by genetic predispositions and lifestyle-related factors.

Muscular strength is defined as the maximum force produced by a muscle or muscle group against resistance (Suchomel et al., 2018). In this study, PA was found to positively support muscular strength. The findings are consistent with evidence indicating that regular PA enhances muscular strength and endurance, thereby improving PF, with this effect being particularly pronounced during adolescence (Bogdanis, 2012; Hughes et al., 2018; Kraav et al., 2022; Koźlenia et al., 2024). This association may be explained through neuromuscular adaptations (Enoka and Duchateau, 2008) as well as muscle protein synthesis and exercise-induced hormonal responses (testosterone, GH, IGF-1) (Kraemer and Ratamess, 2005; Schoenfeld, 2010). Nevertheless, muscular strength may also be influenced by factors such as training intensity, genetic characteristics, and nutritional status.

Agility refers to the ability to change direction rapidly, maintain balance, and minimize reaction time (Sheppard and Young, 2006). The literature indicates that PA supports the development of agility (Young et al., 2015; Thieschäfer and Büsch, 2022), and the present study likewise found that regular PA was associated with higher agility levels, which were in turn related to overall PF. This effect may be explained by neuromuscular adaptations and motor control processes within the central nervous system (Brughelli et al., 2008). However, changes in agility may also be influenced by training type, motor learning, and individual neuromuscular coordination.

### 4.6 PF and working memory

The present study found a significant association between PF level and working memory. This finding is consistent with prior research reporting superior cognitive performance among individuals with higher PF levels (Lambourne, 2006; Chaire et al., 2020; Zhidong et al., 2021; Shi et al., 2022). PF may support working memory performance by enhancing cerebral oxygen delivery and circulatory efficiency. However, this relationship is not limited to physiological improvements alone and may also develop through interactions with cognitive processes such as attention, self-regulation, and mental resilience.

### 4.7 Working memory and mathematics achievement

Working memory is closely associated with mathematics achievement (Raghubar et al., 2010), and individuals with higher achievement have been shown to exhibit advantages particularly in visual–spatial working memory (Bakker et al., 2023). Through the phonological loop, visuospatial sketchpad, and central executive components, working memory plays a fundamental role in managing cognitive load during arithmetic processing and problem solving (Baddeley, 2012; Alloway and Alloway, 2013). Accordingly, the use of instructional strategies that support working memory is recommended in mathematics education (Sánchez-Pérez et al., 2018; Ei and Oo, 2023).

### 4.8 The mediating role of PF

Physical fitness was found to partially mediate the relationship between physical activity and working memory; however, the magnitude of this mediation was modest (VAF = 0.21). This finding indicates that physical activity maintains a substantial direct association with working memory, while physical fitness provides an additional but limited explanatory contribution. Previous studies have reported that physical fitness is positively associated with cognitive functioning, particularly working memory, in adolescents (Williams et al., 2020; Weber et al., 2021; Zhang et al., 2022; Li et al., 2024; Kjellenberg et al., 2024). Higher fitness levels have been linked to better working memory performance and faster response times in information processing and inhibitory control tasks (Zhang et al., 2022; Williams et al., 2020), and improvements in cardiorespiratory fitness have been associated with enhanced working memory performance (Weber et al., 2021). Accordingly, physical fitness should not be interpreted as the primary or exclusive mechanism linking physical activity to cognitive performance. Rather, it appears to function as one of several physiological pathways—potentially involving improved cerebral circulation and metabolic support—that may contribute to executive functioning alongside the direct effects of physical activity. The magnitude of this indirect pathway may vary depending on individual characteristics, training patterns, and lifestyle factors.

The primary limitation of this study is its cross-sectional design, which restricts causal interpretation of the findings. In addition, PA was assessed using the IPAQ short form, a self-report measure that may be subject to social desirability and recall bias among adolescents, potentially leading to overestimation of PA levels. Therefore, future studies are recommended to assess PA using objective measurement tools.

Future research should employ longitudinal designs to examine the effects of regular PA on mental health, health behaviors, and quality of life over time, thereby providing clearer insight into long-term benefits and potential outcomes. In addition, more diverse samples encompassing different age groups and cultural backgrounds may contribute to a more comprehensive understanding of the relationships between PA, quality of life, and health outcomes. Finally, investigating moderating variables such as social support, environmental conditions, and genetic predispositions may facilitate the development of more targeted and explanatory models of the PA–health relationship.

Several limitations should be considered when interpreting the findings. First, the study was conducted within a single metropolitan area and a specific cultural and educational context. Sociocultural characteristics and the structure of the national education system may influence both physical activity behaviors and academic expectations; therefore, the results may not be fully generalizable to adolescents from different countries, rural settings, or alternative educational systems. In addition, although stratified sampling was implemented, all participants were recruited from schools within the same city, and extreme socioeconomic groups may have been underrepresented. This restricted variability may have reduced the sensitivity of the model to detect SES-related associations. Second, despite including key behavioral and physiological variables, several potentially relevant confounders were not directly measured. Factors such as sleep duration and quality, nutritional status, psychological motivation, and academic engagement could influence both physical activity and cognitive performance and may partially explain the observed relationships. Third, physical activity was assessed using a validated self-report questionnaire rather than objective monitoring devices. The absence of device-based measurements (e.g., accelerometers) limits the precision of activity quantification and may attenuate the magnitude of associations. Future studies including nationally diverse samples, objective physical activity monitoring, and broader lifestyle indicators would allow a more comprehensive evaluation of the pathways linking physical activity, physical fitness, and cognitive performance.

## 5 Conclusion

In conclusion, this study examined the relationships among socioeconomic status, parental education, physical activity, physical fitness, working memory, and mathematics achievement within an integrated framework. The findings indicate that working memory is strongly associated with mathematics achievement and that physical activity is positively related to physical fitness and working memory. Physical fitness showed a statistically significant but modest mediating contribution in the association between physical activity and working memory.

However, because of the cross-sectional design, the observed relationships should not be interpreted as causal. The results indicate associations rather than directional effects, and it remains possible that reciprocal or unmeasured factors contribute to the observed pathways. Nevertheless, the findings suggest that promoting opportunities for regular physical activity and supporting physical fitness development in school settings may be relevant for cognitive and academic functioning during adolescence. Future longitudinal and experimental studies are required to clarify the causal mechanisms underlying these relationships.

## Data Availability

The raw data supporting the conclusions of this article will be made available by the author, without undue reservation.
