# Supplementary material for: The relationship between physical activity, working memory, and mathematics achievement on the basis of socioeconomic status: the mediating role of physical fitness level
Source: Front Behav Neurosci. 2026 Mar 12;20:1795851. doi: 10.3389/fnbeh.2026.1795851 (PMC13018152; doi:10.3389/fnbeh.2026.1795851)
Supplement: Supplementary file 1 [file Table_1.docx]

**Calculation process and components of the SES variable**

The socioeconomic status (SES) score was calculated using the formula: SES=30.978+(Average Years of Education in the Household×0.775)+(Per Capita Household Income×0.003)+(Home Ownership×1.975)+(Second Home Ownership×1.975)+(Car Ownership×1.975)+(Presence of Natural Gas Heating×1.775)+(Dishwasher Ownership×1.775)+(Second TV Ownership×1.775)+(Subscription to a Digital Platform×1.775)+(Home Internet Connection (WiFi)×1.775)+(Household Occupational Status Score×6.446)

The SES score calculated using this formula was categorized as A (70.0 and above), B (60.00–69.99), C1 (50.00–59.99), C2 (40.00–49.99), and D (30.00–39.99).
