# Supplementary material for: The relationship between physical activity, working memory, and mathematics achievement on the basis of socioeconomic status: the mediating role of physical fitness level
Source: Front Behav Neurosci. 2026 Mar 12;20:1795851. doi: 10.3389/fnbeh.2026.1795851 (PMC13018152; doi:10.3389/fnbeh.2026.1795851)
Supplement: Supplementary file 2 [file Table_2.docx]

**Detailed inspection procedures**

**Cardiovascular Examination**

The cardiovascular system, a fundamental component of physical fitness (PF), was assessed through specific tests. Blood pressure was measured after participants rested for five minutes in a seated position. The cuff was placed 2–3 cm above the upper arm, and appropriate pressure was applied. A stethoscope was positioned over the brachial artery to record systolic (first sound) and diastolic (last sound) blood pressure values, which were evaluated based on WHO's reference ranges for children (de Onis et al., 2007). For pulse measurement, participants were seated comfortably, and pulse counting was performed manually by palpating the radial artery with light pressure for 60 seconds (Gillum, 1991). The recorded values were compared with age-appropriate reference ranges (Ostchega et al., 2011).

**Respiratory System Examination**

The assessment of the respiratory system was essential to prevent potential breathing issues during PF tests. The examination included inspection, palpation, percussion, and auscultation. During the inspection, respiratory movements, chest symmetry, and the muscles used during breathing were evaluated (Walker et al., 1990). Palpation was conducted to assess chest mobility and detect any tissue abnormalities (Bendtsen et al., 1995). Percussion was performed by gently tapping the thorax to determine lung tissue density and identify areas of fullness or emptiness (Narula et al., 2018). Auscultation involves listening to lung sounds using a stethoscope to detect normal vesicular breath sounds and identify any abnormal sounds, such as wheezing or rales (Şengül Emeksiz & Bostancı, 2018).

**Musculoskeletal System Examination**

The musculoskeletal system examination was conducted to assess posture, muscle strength, and joint range of motion, which are key components of physical fitness (PF). In postural analysis, participants' standing posture and body symmetry were observed, and abnormalities such as scoliosis, kyphosis, and lordosis were evaluated (Perry et al., 2008). The joint range of motion was measured using a goniometer for major joints and compared with age-appropriate reference ranges (Reese and Bandy, 2016). Muscle strength was assessed using Manual Muscle Testing (MMT) to evaluate muscular function and force output (Avers and Brown, 2018).

**General Health Examination**

The general health assessment involved collecting and evaluating information about participants' current and past medical conditions. During anamnesis, participants were asked about their medical history, chronic illnesses, allergies, and regular medication use. Based on the findings of the health examination, participants' eligibility for inclusion in the study was determined.

**References**

Avers, D., and Brown, M. (2018). Daniels and Worthingham's Muscle Testing, First South Asia Edition E-Book: Techniques of Manual Examination and Performance Testing. Elsevier.

Bendtsen, L., Jensen, R., Jensen, N. K., and Olesen, J. (1995). Pressure-controlled palpation: a new technique which increases the reliability of manual palpation. Cephalalgia : an international journal of headache, 15(3), 205–210. doi: 10.1046/j.1468-2982.1995.015003205.x

de Onis, M., Onyango, A. W., Borghi, E., Siyam, A., Nishida, C., and Siekmann, J. (2007). Development of a WHO growth reference for school-aged children and adolescents. Bulletin of the World Health Organization, 85(9), 660–667. [doi: 10.2471/blt.07.043497](https://doi.org/10.2471/blt.07.043497)

Gillum R. F. (1991). Resting pulse rate of children and young adults associated with blood pressure and other cardiovascular risk factors. Public health reports (Washington, D.C. : 1974), 106(4), 400–410.

Narula, J., Chandrashekhar, Y., and Braunwald, E. (2018). Time to Add a Fifth Pillar to Bedside Physical Examination: Inspection, Palpation, Percussion, Auscultation, and Insonation. JAMA cardiology, 3(4), 346–350. doi: 10.1001/jamacardio.2018.0001

Ostchega, Y., Porter, K. S., Hughes, J., Dillon, C. F., and Nwankwo, T. (2011). Resting pulse rate reference data for children, adolescents, and adults: United States, 1999-2008. National health statistics reports, (41), 1–16.

Perry, M., Smith, A., Straker, L., Coleman, J., and O’Sullivan, P. (2008). Reliability of sagittal photographic spinal posture assessment in adolescents. Advances in Physiotherapy, 10(2), 66–75. doi: 10.1080/14038190701728251

Reese, N. B., and Bandy, W. D. (2016). Joint range of motion and muscle length testing-E-book. Elsevier.

Şengül Emeksiz, Z., and Bostancı, İ. (2018). Akciğer: Sesimi Duyan Var Mı? Güncel Pediatri, 16(3), 79-84. doi: 10.32941/pediatri.485522

Walker, H. K., Hall, W. D., and Hurst, J. W. (Eds.). (1990). Clinical Methods: The History, Physical, and Laboratory Examinations. (3rd ed.). Butterworths.
